# Chemerin alleviates the placental oxidative stress and improves fetal overgrowth of gestational diabetes mellitus mice induced by high fat diet

**DOI:** 10.1186/s10020-024-01007-2

**Published:** 2024-11-30

**Authors:** Xuan Zhou, Yi Jiang, Zizhuo Wang, Lijie Wei, Huiting Zhang, Chenyun Fang, Shenglan Zhu, Yuanyuan Du, Rui Su, Weikun Li, Zhenzhen He, Liangnan Zhang, Weidong Tan, Mengzhou He, Jun Yu, Shaoshuai Wang, Wencheng Ding, Ling Feng

**Affiliations:** Department of Obstetrics and Gynecology, Tongji Hospital, Tongji Medical College, Huazhong University of Science and Technology, Wuhan, 430030 People’s Republic of China

**Keywords:** Gestational diabetes mellitus, Animal model, Chemerin, Oxidative stress, Placenta, Fetal growth

## Abstract

**Background:**

Evidence has shown that oxidative stress induced by high glucose microenvironment in placenta of gestational diabetes mellitus (GDM) is indispensable to the progression of this condition. Adipokine chemerin was linked with GDM, yet the roles of chemerin in placental oxidative stress and its underlying effects on GDM in vivo remain elusive.

**Methods:**

We firstly analyzed the disparities of oxidative stress levels in placenta between GDM and normoglycaemic pregnant women, and then added recombinant active chemerin to the high-glucose treated human trophoblastic cells to investigate effects of chemerin on reactive oxygen species (ROS), total antioxidant capacity (T-AOC) and intake of glucose. Finally, a GDM animal model induced by high-fat diet (HFD) was established and the impacts of chemerin on oxidative stress of placenta and fetal growth of GDM were explored.

**Results:**

Analysis of human samples showed that the extent of lipid peroxidation in placenta was significantly elevated in GDM patients compared with their normoglycaemic counterparts. In the high glucose cell model, active chemerin lessened the content of ROS, heightened the index of T-AOC and stimulated glucose uptake in a concentration-dependent manner. Importantly, we successfully constructed a GDM mouse model through HFD. The treatment of chemerin was found to alleviate the high blood glucose levels in these HFD-fed pregnant mice and attenuate the excessive growth of their offspring. Our data also revealed that chemerin might counteract placental oxidative stress in HFD mice by improving the activity of superoxide dismutase.

**Conclusions:**

The present study further elucidated the molecular biology of chemerin, which plays a pivotal role in ameliorating oxidative stress and hyperglycemia, resulting in improved fetal overgrowth in GDM.

**Graphical abstract:**

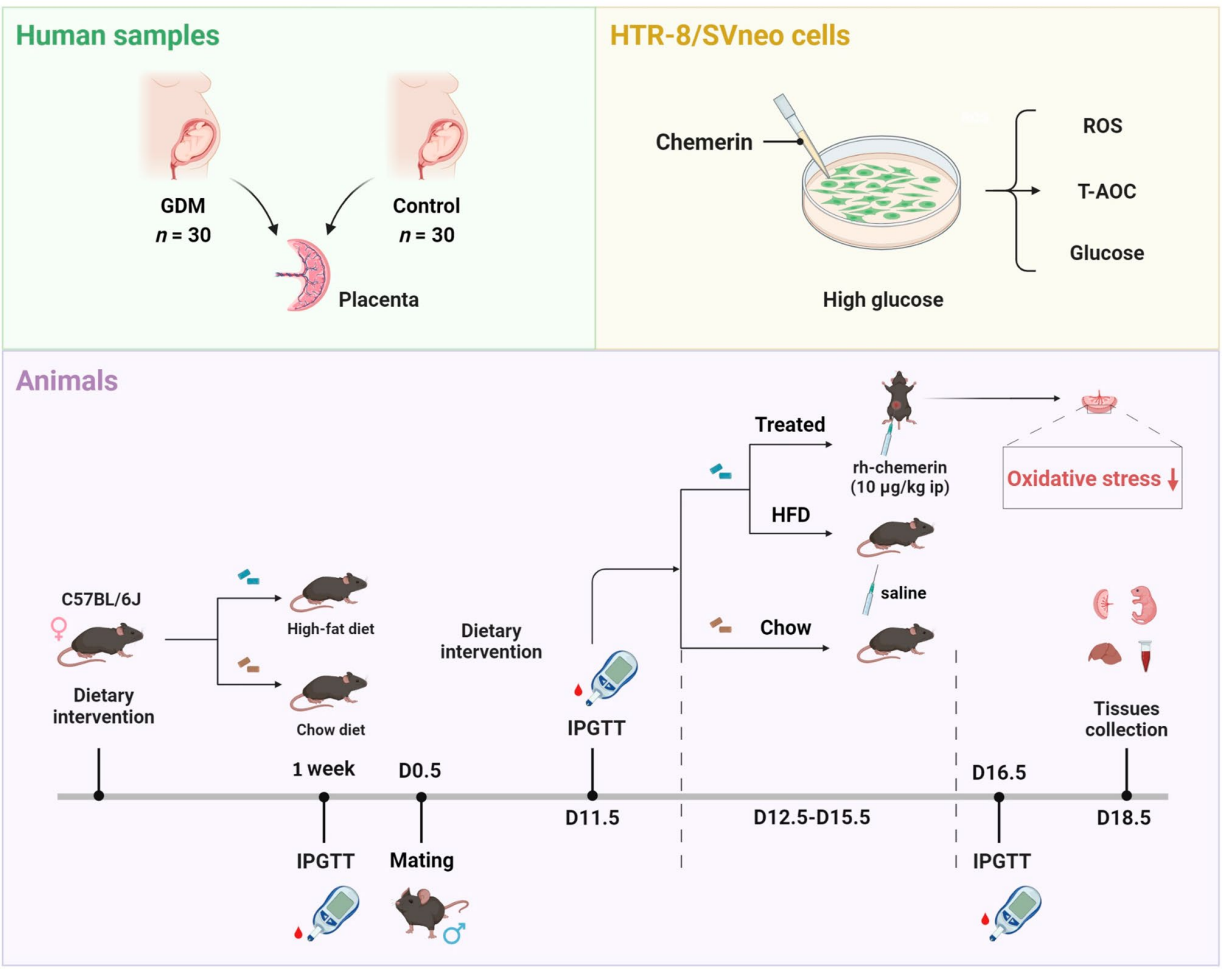

## Introduction

Gestational diabetes mellitus (GDM) is a special type of diabetes associated with adverse pregnancy outcomes (Ye et al. 2022; Vounzoulaki et al. 2020). The global prevalence of GDM ranged from 9% to 26% (Jiang et al. 2022). Strong evidence suggests that pathological insulin resistance of GDM leads to abnormal glycometabolism and elevated glucose concentration in the placenta. High glucose could contribute to increased production of reactive oxygen species (ROS), which subsequently triggers mitochondrial dysfunction and oxidative stress, further exacerbating insulin resistance (Cojocaru et al. 2023; Saucedo et al. 2023). The topic of placental oxidative stress is currently attracting considerable interest as a prospective factor inducing GDM, though the specific mechanisms involved remain to be fully elucidated.

Chemerin is a novel adipokine and encoded by retinoic acid receptor responder 2 (*RARRES2*) gene (Ruszała et al. 2021). Its inactive precursor requires extracellular proteolytic cleavages at C-terminal to generate various biological activities, of which chem157S is the highest active form (Zhao et al. 2022). Our previous research found that chemerin could be secreted by placenta and its expression was markedly elevated in GDM placenta compared to the normoglycaemic pregnant women (Zhou et al. 2023). Cell experiments found that treatment of recombinant active chemerin was beneficial to the enhanced conduction of the phosphatidylinositol 3-kinase (PI3K)-protein kinase B (AKT/PKB) pathway, known as a critical insulin signaling pathway (Zhou et al. 2020a; Lennicke and Cochemé 2021).

As a mitochondria-localized chaperone protein, disulfide-bond A oxidoreductase-like protein (DsbA-L) was believed to exert a key influence on maintaining mitochondrial function and homeostasis (Zhou et al. 2021). The deletion of DsbA-L could impair mitochondrial functions and active the cyclic GMP-AMP synthase (cGAS)-stimulator of interferon genes (STING) signaling pathway, exacerbating inflammation and insulin resistance (Bai et al. 2020; Oduro et al. 2022). Evidence had shown that cGAS-STING pathway was inextricably linked with metabolism (Bai and Liu 2019). Our earlier study demonstrated that in contrast with the normoglycaemic pregnant women, the expression of DsbA-L was evidently decreased, and the morphology of mitochondria was relatively swollen in GDM placenta. Meanwhile, data revealed that the activation of cGAS-STING pathway might play an important effect on the development of GDM (Zhou et al. 2023).

Previous experiments in vitro highlighted the pivotal role of chemerin in promoting the expression of DsbA-L and regulating the cGAS-STING pathway in human trophoblast cells (Zhou et al. 2020b, 2023). Animal studies have confirmed that chemerin possesses the capability to augment superoxide dismutase (SOD) activity and reduce the content of malondialdehyde (MDA) (An et al. 2020). In addition, the activation of ChemR23, receptor of chemerin was identified to have a role of ameliorating oxidative stress (Zhang et al. 2022). Nevertheless, the potential effects and mechanisms of chemerin on GDM placental oxidative stress in vivo requires further investigation.

In this study, we analyzed the discrepancy of placental oxidative stress between GDM and normoglycaemic pregnant women, and assessed the contributions of chemerin in enhancing ROS clearance and augmenting total antioxidant capacity (T-AOC). To delve deeper into the roles of chemerin in GDM, we devised a GDM mouse model through the administration of a high-fat diet, and subsequently validated the molecular mechanisms underlying the effects of chemerin on placental oxidative stress and insulin resistance in vivo. The findings of this study offer invaluable insights into the therapeutic targets of GDM and may serve as a guiding framework for future research endeavors.

## Materials and methods

### Clinical samples collection

A total of 60 pregnant women from Tongji Hospital, Tongji Medical College, Huazhong University of Science and Technology, were enrolled in the study. These participants comprised 30 cases of GDM and 30 cases with normal glucose tolerance (designated as control). GDM were diagnosed at 24 ~ 28 weeks of gestation through the 75 g oral glucose tolerance test (OGTT) according to the standards of International Association of Diabetes and Pregnancy Study Groups. The participants were all singleton pregnancies at term labor without any additional complications during pregnancy. Following cesarean deliveries, samples of placenta tissues were collected immediately. This research endeavor was granted approval by the Ethics Committee of Tongji Hospital (TJ-IRB20220104), and prior informed consent was secured from each individual participant.

### Antioxidant enzymes and oxidative level assay

The activities of SOD (KTB1030), glutathione peroxidase (GSH-Px, KTB1640) and catalase (CAT, KTB1040), the content of MDA (KTB1050) in placenta tissues were separately assayed with commercially available kits (Abbkine Scientific Co., Ltd, China), and lipid peroxidation (LPO, A106-1) as well as oxidized glutathione (GSSG, A061-1) were detected using the assay kits from Nanjing Jiancheng Bioengineering Institute, China, according to the manufacturer’s instructions.

### Cell culture and treatment

Human trophoblast originated HTR-8/SVneo cell lines (obtained from Servicebio, China) were initially cultured in RPMI 1640 medium (Gibco, Thermo Fisher Scientific, USA) containing 11 mmol/L glucose, and the medium were supplemented with 10% fetal bovine serum and 1% penicillin/streptomycin. In accordance with the previous researches (Valent et al. 2021; Deng et al. 2023), the RPMI 1640 medium was additionally complemented with D-glucose powder (biofroxx, Germany) to become high glucose medium (HG, 25 mmol/L). HTR-8/SVneo cells were incubated in the above two different glucose concentrations for 48 h at 37℃ in a humidified atmosphere with 5% CO_2_.

### Reactive oxygen species (ROS)

The ROS level was measured using a ROS assay kit (S0033S, Beyotime Biotechnology, China) according to the manufacturer’s protocol. HTR-8/SVneo cells were divided into three groups. Among them, the control group and the HG group were treated by DCFH-DA (10 µmol/L) fluorescence probe for 20 min at 37℃. Cells was incubated with serum-free medium as substitution in the blank group. Finally, the cells were washed 3 times and collected by 0.25% Trypsin-EDTA (C100C1, New Cell & Molecular Biotech, China), and the fluorescence intensity was measured by BioTek Synergy2 multi-mode microplate reader (Gene Company limited, USA).

### Total antioxidant capacity (T-AOC) assay

The antioxidant capacity of cells in different groups were examined using the T-AOC assay kit with a rapid 2,2’-azino-bis (3-ethylbenzothiazoline-6-sulfonic acid) (ABTS) method (S0121, Beyotime Biotechnology, China). Firstly, cells (1 × 10^6^) were collected by scraping directly, and then sonicated to obtain antioxidants at 4℃. Then 10 µL cell samples were mixed gently with 20 µL peroxidase working solution and 170 µL ABTS working solution in the dark. After incubation for 6 min at room temperature, the absorbance was measured at 414 nm. The results were calculated according to the manufacturer’s standard.

### Glucose uptake assay

The concentration of glucose in the cell culture medium was detected by a glucose assay kit with O-toluidine (S0201S, Beyotime Biotechnology, China). Briefly, 10 µL samples and 180 µL glucose assay reagent were mixed by vortex and heated for 8 min at 95℃ in the PCR thermal cycler (Applied Biosystems, USA). After the system was cooled down to 4℃, absorbance was detected using a microplate reader (Thermo Fisher, USA) at 630 nm, and the concentration of glucose was calculated according to the standard curve. The amount of glucose consumed by cells was the level of remaining glucose subtracted from the initial glucose content.

### Establishment of experimental mouse model

The animal experimental protocols were approved by the Ethics Committee for Laboratory Animal Welfare of Tongji Hospital, Tongji Medical College, Huazhong University of Science and Technology (TJH-202110011). A total of 60 healthy female C57BL/6J mice and 30 healthy male C57BL/6J mice were obtained from Beijing HFK Bioscience Co., Ltd. All mice were 6-weeks-old and lived in specific pathogen free (SPF) barrier facilities with room temperature, appropriate humidity (50%~60%) and a 12 h light/dark cycle. After one week of adaptive feeding, the body weight of all mice was measured.

As previously validated (Fang et al. 2023; Tang et al. 2023; Li et al. 2023), the female mice were randomly divided into two groups, one group was fed high fat diet (HFD, 60 kcal% fat, Research Diets #D12492, USA), the other group was access to a regular chow diet. The body weight of female mice was recorded daily, and after one week of feeding, the female mice were bred with male mice in a 2:1 ratio, and gestation day (D) 0.5 was considered as the day on which the vaginal mucous plug was observed. The pregnant mice of HFD group were maintained with high fat diet during the whole pregnancy, while the control group were continually fed with chow diet.

### Intraperitoneal glucose tolerance test (IPGTT) and chemerin treatment

The IPGTT was performed firstly before mice mating, and the second time in pregnant mice on the D11.5, with an intraperitoneal injection of 20% glucose solution (2 g/kg body weight) after 14 ~ 16 h fasting. Blood glucose levels were measured at the specified time points (0、30 min、60 min、90 min、120 min) with a glucometer (Accu-Chek Performa, Roche Diagnostic, Germany).

Afterward, the HFD pregnant mice were equated the treated group and the non-treated group. For treatment, pregnant mice were intraperitoneally injected the active recombinant human chemerin (10 µg/kg, HY-P70099, MedChemExpress, USA) dissolved in 200 µL saline every day during D12.5∼D15.5, while mice of the other group was injected intraperitoneally with 200 µL saline as control. Blood glucose levels of all pregnant mice (the chow group, the HFD group and the treated group) were assessed the third time by IPGTT on the D16.5, and specimens such as placentas, fetal mice, liver tissues, and orbital venous blood samples were taken for subsequent experiments on the D18.5. Several pregnant mice gave birth without intervention and were fed with normal chow diet until 8 weeks postpartum and obtained samples.

### Transmission electron microscope (TEM)

The placental tissues fixed in 2.5% cold glutaraldehyde for 24 h and in 1% osmic acid at room temperature for 2 h were sequentially dehydrated by gradient ethanol for 20 min every times. And the epoxy resin was used to permeate and embed the placenta, which were sliced into ultrathin sections next. Finally, the sections were stained by 2% uranyl acetate and lead citrate respectively for 15 min, followed by being dried at room temperature overnight. The TEM was used to observe the structure of mitochondria and acquire images on the next day.

### Hematoxylin eosin and oil red O staining

After fixation in 4% paraformaldehyde at room temperature for approximately 24 h, the harvested liver tissues of mice were embedded in paraffin and cut into sections. Then, the paraffin wax slices were subjected to hematoxylin eosin (HE) staining in accordance with standard protocols. After the samples were sealed with neutral balsam, digital images were captured using a scanner.

In addition, other liver samples were dehydrated in 30% sucrose and then embedded by optimal cutting temperature compound. For oil red O staining, 8-µm-thick sections were prepared using a freezing microtome. The sections were then stained by oil red O solution protected from light for approximately 10 min. After differentiation by 75% ethanol and nuclei being stained in hematoxylin for 5 min, the slices were sealed with glycerol gelatin (Servicebio, China) and observed under the microscope.

### Immunohistochemical analysis

The paraffin-embedded slices of placenta were firstly removed paraffin and rehydrated, then they were quenched in 3% hydrogen peroxide for 30 min at 37℃. After the sections were performed with heat-induced antigen retrieval and blocked by 5% bovine serum albumin for 30 min at 37℃, they were incubated overnight at 4°C with the following antibodies: RARRES2/chemerin rabbit antibody (1:200, A6963, ABclonal); DsbA-L rabbit antibody (1:200, 14535-1-AP, Proteintech). The negative control sections were prepared by substitution of the primary antibodies with phosphate-buffered saline. The horseradish peroxidase-conjugated anti-rabbit IgG antibody and 3,3’-diaminobenzidine (DAB) chromogen were applied successively on the sections on the next day. Lastly, the sections were examined under the scanner (Shenzhen Shengqiang Technology, China), and the quantitative analysis was performed by Image-Pro Plus 6.0 (Media Cybernetics, Denver, USA).

### Enzyme-linked immunosorbent assay (ELISA) and biochemical analysis

The serum of orbital venous blood from pregnant mice were collected for analysis. Fasting insulin levels were measured using a mouse insulin ELISA kit (P01325, RayBiotech Life, USA) and the content of chemerin was assayed by the mouse chemerin ELISA kit (EK1330, Boster Biological Technology, China), assays were both according to the manufacturer’s protocols.

The fasting serum levels of alanine aminotransferase (ALT), aspartate transaminase (AST), high-density lipoprotein (HDL), low-density lipoprotein (LDL), total cholesterol (TC) and triglyceride (TG) of pregnant mice were detected using the automatic biochemistry analyzer, respectively.

### Western blotting

Proteins of placenta tissues were extracted from pregnant mice at 4℃ and their concentrations were measured by bicinchoninic acid method. Then denatured proteins were separated by SDS-PAGE gel (P0012A, Beyotime Biotechnology, China) electrophoresis before transferring to polyvinylidene fluoride (PVDF) membranes. After the membranes were blocked by 5% bovine serum albumin for 1 h at room temperature, the following primary antibodies were incubated with PVDF membranes overnight at 4 °C, including anti-RARRES2/chemerin (1:2000, A6963, ABclonal), anti-DsbA-L (1:2000, ab92819, Abcam), anti-IRS1 (1:1000, A0245, ABclonal), anti-PI3K p110β (1:1000, ab151549, Abcam), anti-AKT2 (1:5000, ab131168, Abcam), anti-STING (1:2000, 19851-1-AP, Proteintech), anti-TBK1 (1:5000, ab40676, Abcam), anti-IRF3 (1:1000, ab68481, Abcam), anti-phospho-TBK1 (Ser172) (1:1000, 5483T, CST), anti-phospho-IRF3 (Ser386) (1:1000, 37829T, CST), anti-β-actin (high dilution) (1:10000, AC026, Abclonal). On the following day, membranes were washed and incubated with secondary antibodies for 1 h at room temperature. Thereafter, protein bands were visualised using enhanced chemiluminescence reagents (BMU102, Abbkine) and images were captured by the device of G: BOX Chemi XRQ (Syngene, UK). The signal intensity of detected bands were analyzed using Image J software (NIH, USA).

### Real-time quantitative PCR analysis

The total RNA of placenta tissues were extracted using RNAiso Plus (#9109, Takara, Japan) and RNase free Eppendorf tubes at 4℃. HiScript II qRT SuperMix (R222-01, Vazyme, China) was used for reverse transcription in PCR thermal cycler (Applied Biosystems, USA). Then the synthesized cDNA and primers were mixed with ChamQ Universal SYBR qPCR Master Mix (Q711-02, Vazyme, China) and amplified in CFX96 Touch Real-time PCR Detection System (Bio-Rad, USA). The sequences of the primers used are presented in Table 1. The relative expressions of mRNA of target genes were normalized with *β-actin* gene and the results were quantified using the 2^−ΔΔCt^ method.

**Table 1:** Sequences of the primers

Gene Name	Primer Sequence
*Mouse TBK1*	Forward 5’-GACATGCCTCTCTCCTGTAGTC-3’
*Mouse TBK1*	Reverse 5’-GGTGAAGCACATCACTGGTCTC-3’
*Mouse IRF3*	Forward 5’-CGGAAAGAAGTGTTGCGGTTAGC-3’
*Mouse IRF3*	Reverse 5’-CAGGCTGCTTTTGCCATTGGTG-3’
*Mouse β-actin*	Forward 5’-CATTGCTGACAGGATGCAGAAGG-3’
*Mouse β-actin*	Reverse 5’-TGCTGGAAGGTGGACAGTGAGG-3’

### Statistical analysis

Statistical analyses were performed using SPSS 25.0 (Chicago, IL) software. Comparisons between two groups were performed by the unpaired Student’s *t*-test after validating normality and the continuous variables were expressed as mean ± standard deviation (SD). Graphs were plotted using GraphPad Prism 9.0 (San Diego, CA) and typeset by Adobe Photoshop. All experiments were conducted independently at least three times. *P* value < 0.05 was considered statistically significant.

## Results

### Clinical basic characteristics of pregnant women

The clinical basic characteristics of participants in the study were summarized in Table 2. Upon statistical analysis, no significant variations were observed in maternal age, pre-pregnancy body mass index (BMI) and gestational age at delivery of pregnant women between the two groups. Nevertheless, it is noteworthy that the blood glucose levels measured during OGTT in the GDM group were conspicuously elevated compared to those recorded in the control group.

**Table 2:** Clinical basic characteristics

	GDM (*n* = 30)	Control (*n* = 30)	*P* value	t value
Maternal age (years)	31.93 ± 3.26	30.97 ± 3.47	0.271	-1.113
Pre-pregnancy BMI (kg/m^2^)	21.19 ± 2.01	20.56 ± 2.26	0.259	-1.140
Gestational age at delivery (days)	270.93 ± 6.51	271.37 ± 5.16	0.776	0.286
OGTT 0 h (mmol/L)	4.90 ± 0.46	4.56 ± 0.26	0.001^**^	-3.566
OGTT 1 h (mmol/L)	9.96 ± 1.15	8.18 ± 1.09	< 0.001^***^	-6.163
OGTT 2 h (mmol/L)	8.41 ± 1.63	6.60 ± 1.14	< 0.001^***^	-4.990

GDM: Gestational diabetes mellitus; BMI: Body mass index; OGTT: Oral glucose tolerance test. All data are expressed as mean ± SD. ^**^P < 0.01; ^***^P < 0.001

### The level of lipid peroxidation was increased in placenta of GDM patients

**Fig. 1:**
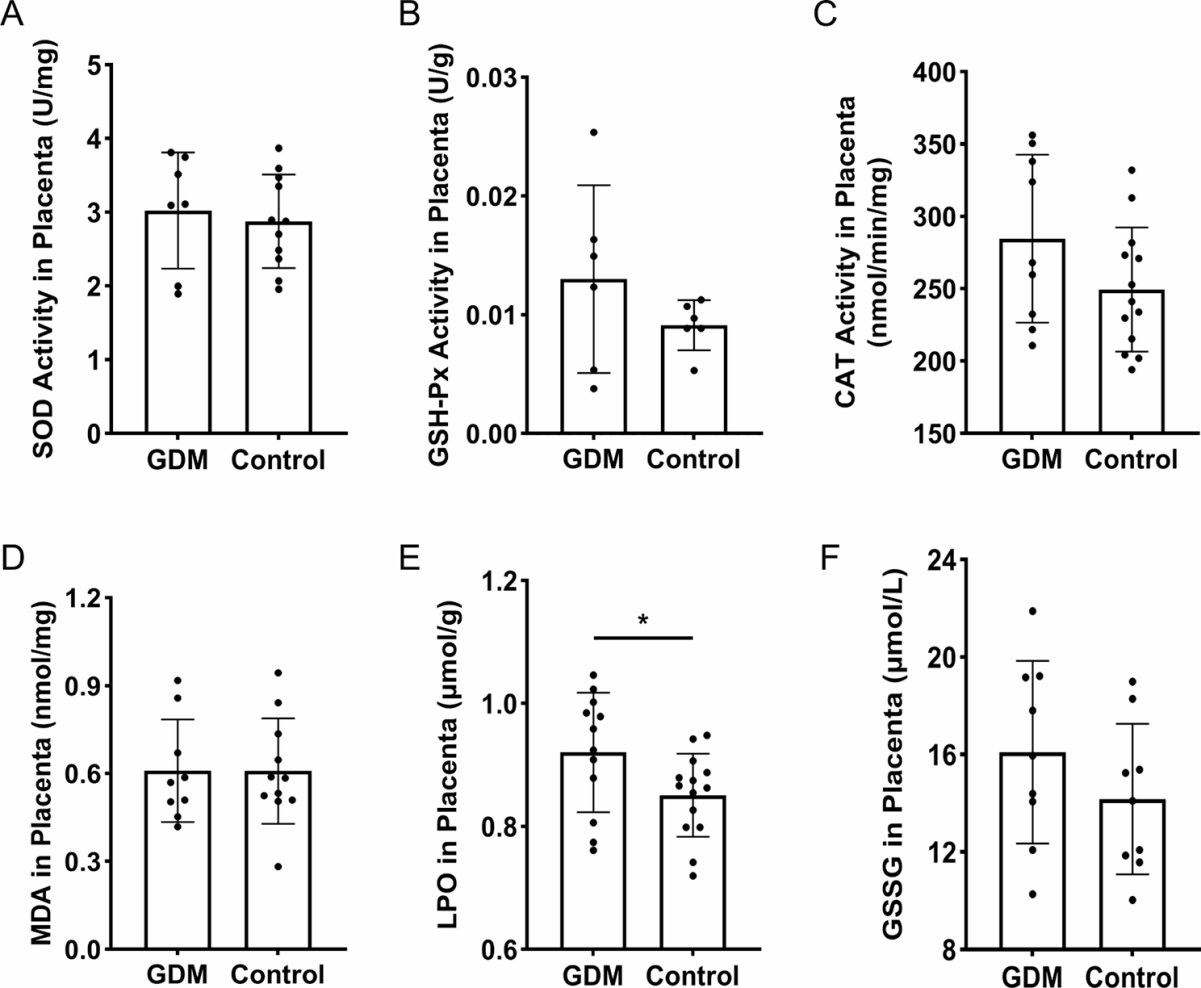
The levels of oxidative stress and activities of antioxidant enzymes in placenta of GDM patients and normoglycaemic pregnant women. (**A**) The activity of SOD in placenta. (**B**) The activity of GSH-Px in placenta. (**C**) The activity of CAT in placenta. (**D**) The content of MDA in placenta. (**E**) The content of LPO in placenta. (**F**) The content of GSSG in placenta. GDM: gestational diabetes mellitus; SOD: superoxide dismutase; GSH-Px: glutathione peroxidase; CAT: catalase; MDA: malondialdehyde; LPO: lipid peroxidation; GSSG: oxidized glutathione. Data were shown as mean ± SD (*n* ≥ 3). ^*^P < 0.05

Oxidative stress is attributed to imbalance between oxidation and antioxidation processes, that is excessive ROS production or insufficient ROS removal. To evaluate the antioxidant capacities of placenta in the GDM group and the control group, the activities of key antioxidant enzymes, including SOD, GSH-Px, CAT were assessed, respectively. Besides, the content of MDA, LPO and GSSG in placenta were separately detected to check oxidative stress level. Our results revealed a slight elevation in the activities of SOD, GSH-Px and CAT enzymes in placenta from GDM group compared to the control group, albeit these differences did not reach statistical significance (Fig. 1A-C). Conversely, the placenta of GDM patients exhibited a significantly higher level of LPO (Fig. 1E, *P* < 0.05), and a trend towards increased GSSG levels, compared to the control group. However, no statistically significant differences were observed in the content of GSSG or MDA in placenta between the two groups of pregnant women (Fig. 1D, F).

### The effects of chemerin on the ROS level, T-AOC and glucose uptake were dependent on its concentrations in high glucose cell model

**Fig. 2:**
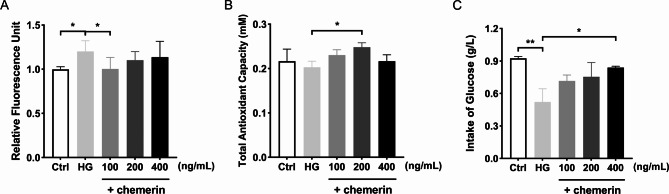
The effects of different concentrations of chemerin on ROS level, T-AOC and glucose uptake in the high glucose cell model. (**A**) The relative fluorescence intensity of ROS. (**B**) The detection of total antioxidant capacity. (**C**) The intake of glucose by cells. HG: high glucose; ROS: reactive oxygen species; T-AOC: total antioxidant capacity. Data were shown as mean ± SD (*n* ≥ 3). ^*^P < 0.05; ^**^P < 0.01

Firstly, a high glucose (HG) cell model was constructed in HTR-8/SVneo cells according to previous methods to simulate GDM placenta in vitro, and the modeling effect has been verified (Zhou et al. 2023). Then, in order to investigate the effects of chemerin on ROS level, T-AOC and glucose uptake, varying concentrations of the active chemerin (100 ng/mL, 200 ng/mL, 400 ng/mL) were administered to HG cell model for 48 h, respectively. Results showed that the ROS production of HG group was significantly higher than that of the control group, while 100 ng/mL chemerin evidently reduced the ROS level when compared with HG group (Fig. 2A), and 200 ng/mL chemerin could considerably enhance the T-AOC of HG cell model (Fig. 2B). Furthermore, the HG group showed decreased intake of glucose when compared to the control group, indicating that there might be insulin resistance in HG group, whereas 400 ng/mL chemerin could obviously promote glucose uptake of cells in HG group (Fig. 2C).

### The establishment of GDM mouse model

**Fig. 3:**
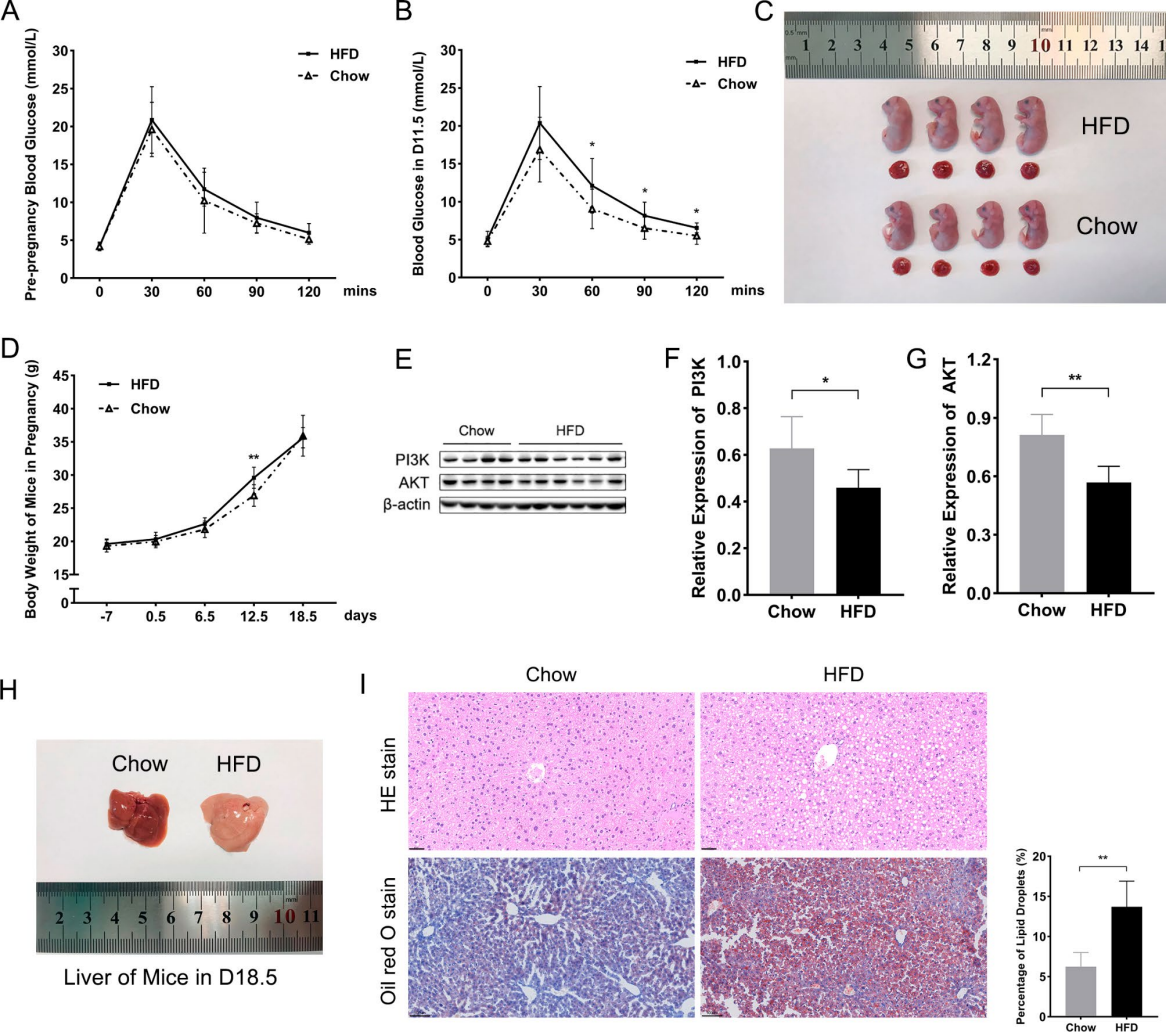
The establishment of GDM mouse model. (**A**) The blood glucose of two groups of mice by IPGTT in pre-pregnancy. (**B**) The blood glucose of two groups of pregnant mice by IPGTT on D11.5. (**C**) The comparisons of sizes of fetal mice and placentas of two groups on D18.5. (**D**) The changes of body weight of mice from one week before pregnancy to the whole pregnancy. (**E**) Western blotting images of mice placentas on D18.5. (**F**) Protein quantification of PI3K relative to β-actin. (**G**) Protein quantification of AKT relative to β-actin. (**H**) The general appearance of liver tissues of pregnant mice on D18.5. (**I**) Staining of HE and oil red O of livers of pregnant mice on D18.5 (scale bar = 100 μm) and quantitative analysis of lipid droplets. HFD: high fat diet; IPGTT: intraperitoneal glucose tolerance test; PI3K: phosphatidylinositol 3-kinase; AKT/PKB: protein kinase B; HE: hematoxylin eosin. Data were shown as mean ± SD (*n* ≥ 3). ^*^P < 0.05; ^**^P < 0.01

Based on the previous researches (Fang et al. 2023; Tang et al. 2023; Li et al. 2023), a GDM model was devised by administering HFD to female C57BL/6J mice for one week prior to mating and throughout the duration of pregnancy. In order to ascertain the pre-pregnancy glucose tolerance levels in mice, the IPGTT was performed via tail vein blood sampling. Results demonstrated that there was no statistically difference on the glucose levels between the HFD group and the chow group (Fig. 3A), nor were there any discernible differences in the body weight before pregnancy between the two groups (Fig. 3D). Consequently, the possibility of diabetes mellitus prior to pregnancy was eliminated.

Thereafter, the female and male mice were mated. The confirmation of mating was indicated by the presence of a vaginal plug and the day was regarded as D0.5. The dietary formula of pregnant mice in two groups maintained the whole pregnancy and IPGTT was conducted again on D11.5. Results showed that the blood glucose levels of HFD mice at 60 min, 90 min, 120 min were all significantly higher than those of chow diet mice (Fig. 3B). This observation suggests that there was an abnormal glucose tolerance in HFD mice. Besides, the average body weight of pregnant mice in HFD group was notably elevated compared with the chow group on the D12.5 (Fig. 3D). On gestation day 18.5, the weight of fetal mice and placentas in HFD group was both visibly higher than the chow group (Figs. 3C and 6B-D). For insulin signaling, results suggested that the protein expressions of PI3K and AKT were markedly decreased in the HFD group compared to the chow group (Fig. 3E-G). Taken together, our data indicated that HFD mice developed insulin resistance (IR) as exhibited by the increased blood glucose levels of IPGTT and impaired insulin signaling.

**Fig. 4:**
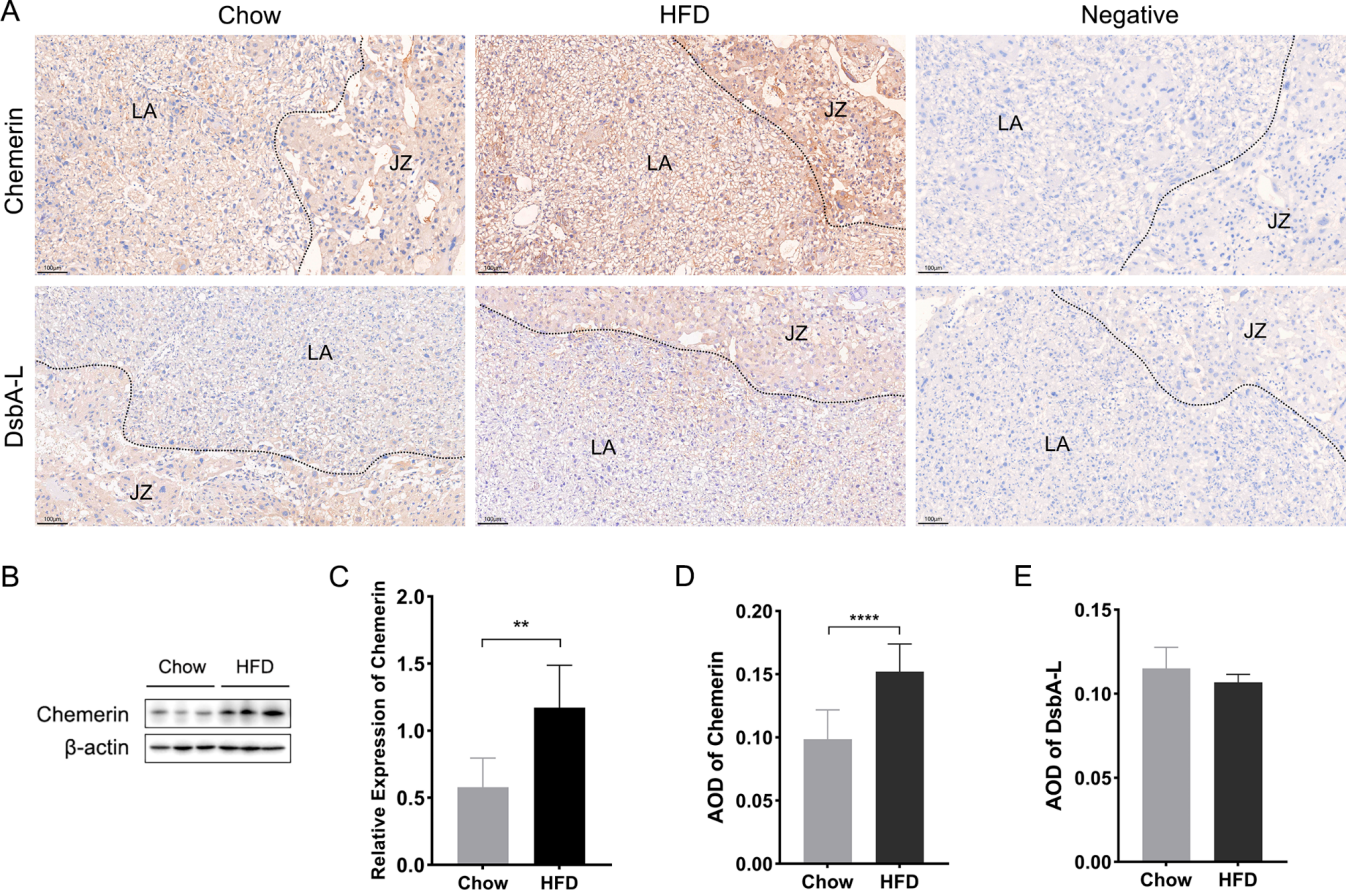
The localization and expressions of chemerin and DsbA-L in mice placenta of the HFD groups and the chow groups. (**A**) Staining of chemerin and DsbA-L by immunohistochemistry (scale bar = 100 μm). (**B**) Western blotting images of mice placentas. (**C**) Protein quantification of chemerin relative to β-actin. (**D**) AOD of chemerin in mice placenta. (**E**) AOD of DsbA-L in mice placenta. DsbA-L: disulfide-bond A oxidoreductase-like protein; HFD: high fat diet; LA: labyrinth; JZ: junctional zone; AOD: average optical density. Data were shown as mean ± SD (*n* ≥ 3). ^**^P < 0.01, ^****^P < 0.0001

As shown in the Fig. 3H, the color of mice livers in the HFD group turned white visibly compared to the chow group. To check the hepatic degeneration and morphological changes in pregnant mice, oil red O staining and hematoxylin eosin staining were performed, respectively. Results showed that there were histological structure disorders and hepatic steatosis accompanied with obvious fat vacuoles in the HFD mice livers (Fig. 3I).

### Localization and expressions of chemerin and DsbA-L in mice placenta

In order to determine the localization of chemerin and DsbA-L in mice placenta tissues, immunohistochemical experiments were performed. Results showed that chemerin was expressed in the spongiotrophoblasts and labyrinth zone of mice placenta, and DsbA-L was mainly located in junctional zone of placenta (Fig. 4A). Furthermore, results of western blot experiments illustrated that in comparison with the chow group, the expression of chemerin in placenta was notably upregulated in the HFD group (Fig. 4B, C) and analysis of AOD showed the same results (Fig. 4D). However, the differences of DsbA-L presented by ‌immunohistochemistry in the two groups did not have statistical significance (Fig. 4E).

**Fig. 5:**
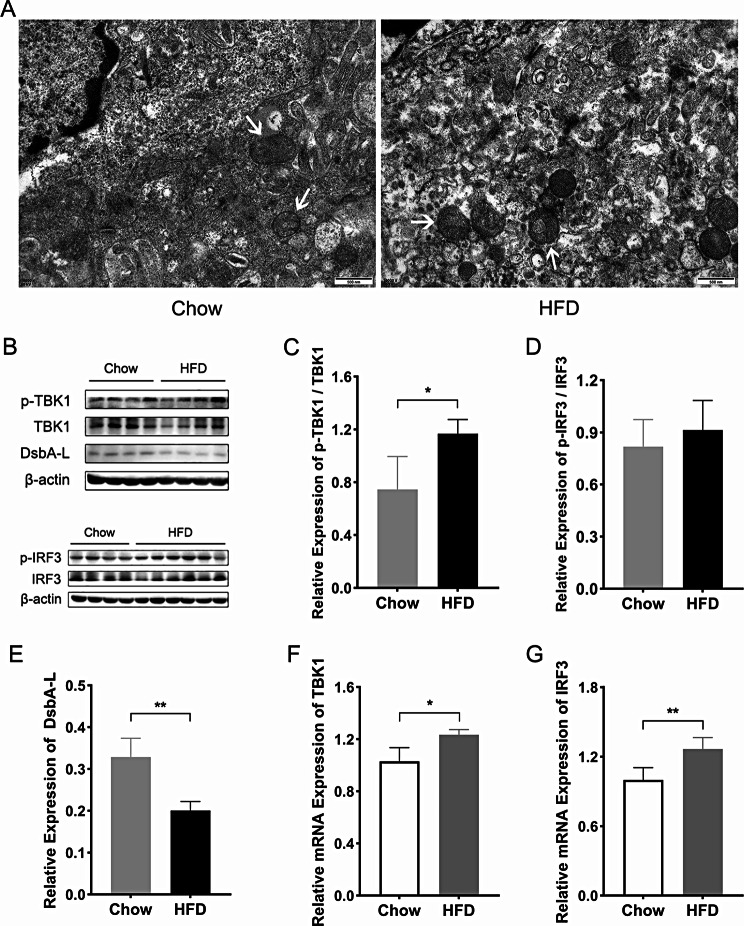
The mitochondrial morphology and the expression levels of DsbA-L, TBK1 and IRF3 in the mice placenta. (**A**) Images of mice placental mitochondria observed by TEM (scale bar = 500 nm). (**B**) Western blotting images of mice placentas. (**C**) Protein quantification of phospho-TBK1 relative to TBK1. (**D**) Protein quantification of phospho-IRF3 relative to IRF3. (**E**) Protein quantification of DsbA-L relative to β-actin. (**F**) The mRNA level of *TBK1* relative to *β-actin*. (**G**) The mRNA level of *IRF3* relative to *β-actin*. White arrows indicated placental mitochondria. HFD: high fat diet; DsbA-L: disulfide-bond A oxidoreductase-like protein; TBK1: TANK binding kinase 1; IRF3: interferon regulatory factor 3. Data were shown as mean ± SD (*n* ≥ 3). ^*^P < 0.05; ^**^P < 0.01

### The mitochondrial morphology, expressions of DsbA-L and the downstream molecules of cGAS-STING pathway in mice placenta

To examine the mitochondrial structure of mice placental trophoblast, the transmission electron microscope was applied. Results indicated the presence of vacuolar changes and cristae structure disorders in placental mitochondria of HFD mice, compared to the mice with chow diet (Fig. 5A). As mitochondria-localized protein, DsbA-L and the downstream molecules of cGAS-STING pathway, TANK binding kinase 1 (TBK1) and interferon regulatory factor 3 (IRF3) in the mice placenta were examined, respectively. Results of western blotting demonstrated that the protein expression of DsbA-L was obviously downregulated and the phospho expression of TBK1 was distinctively higher in the HFD group than those of the chow group (Fig. 5B, C, E). Moreover, the mRNA levels of *TBK1* and *IRF3* in placenta were both significantly increased in the HFD group compared with the chow group (Fig. 5F, G).

**Fig. 6:**
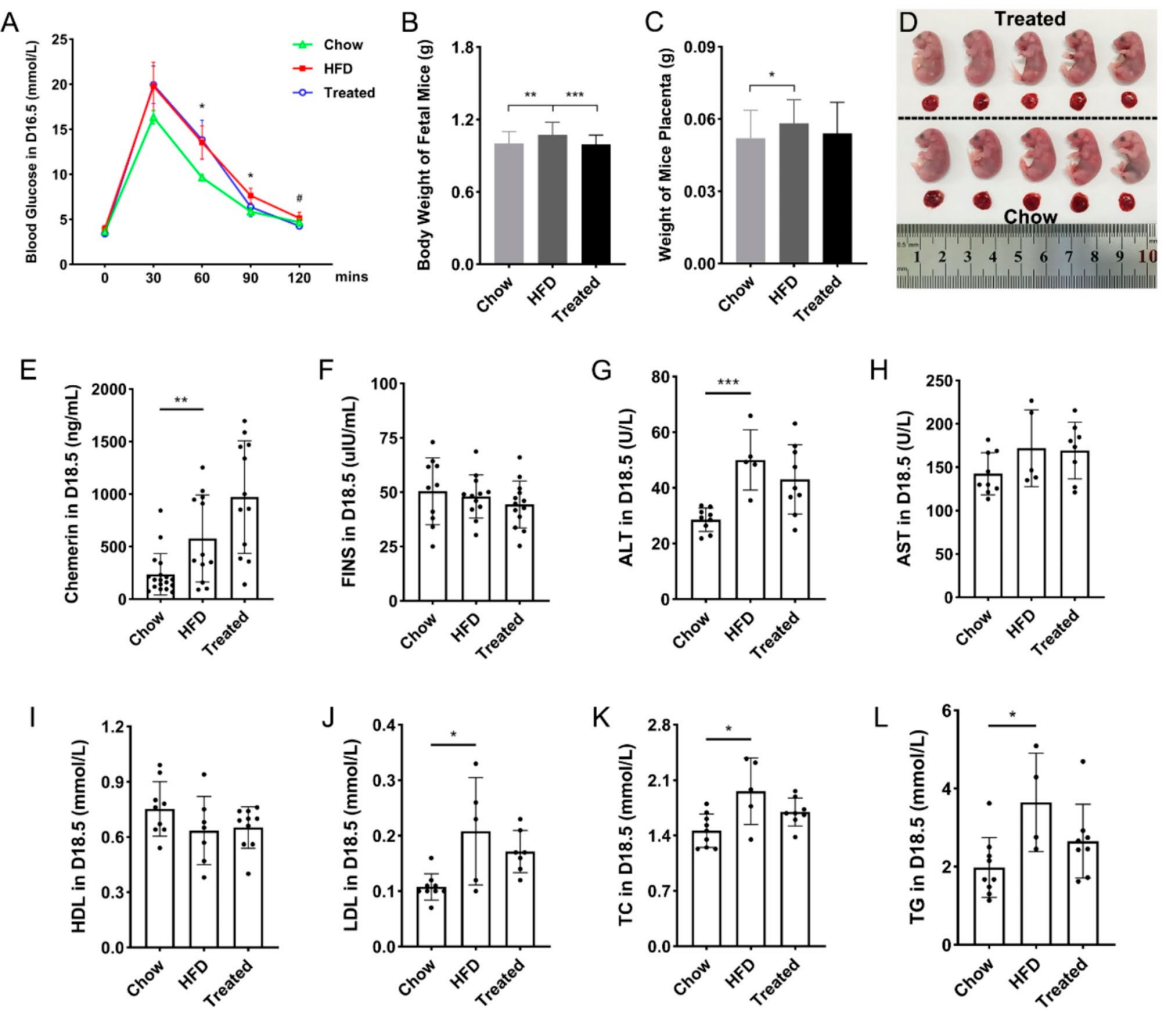
The effects of chemerin on the glucose tolerance, weight of fetal mice and placentas, fasting insulin levels, lipid metabolism and liver function. (**A**) The blood glucose of pregnant mice detected by IPGTT on D16.5 (* represents the comparison between the chow groups and the HFD groups; # represents the comparison between the HFD groups and the treated groups). (**B**) The body weight of fetal mice on D18.5. (**C**) The weight of mice placentas on D18.5. (**D**) The sizes of fetal mice and placentas on D18.5 after chemerin treatment. (**E**) The serum level of chemerin on D18.5. (**F**) The serum level of fasting insulin on D18.5. (**G**) The serum level of ALT on D18.5. (**H**) The serum level of AST on D18.5. (**I**) The serum level of HDL on D18.5. (**J**) The serum level of LDL on D18.5. (**K**) The serum level of TC on D18.5. (**L**) The serum level of TG on D18.5. HFD: high fat diet; FINS: fasting insulin; ALT: alanine aminotransferase; AST: aspartate transaminase; HDL: high-density lipoprotein; LDL: low-density lipoprotein; TC: total cholesterol; TG: triglyceride. Data were shown as mean ± SD (*n* ≥ 3). ^#^*P* < 0.05; ^*^P < 0.05; ^**^P < 0.01; ^***^P < 0.001

### Chemerin decreased blood glucose levels and exerted a slight influence on the lipid metabolism of HFD mice

Half of the HFD pregnant mice were treated continuously by intraperitoneal injection with the active recombinant chemerin^157S^ during D12.5 ~ D15.5, and IPGTT was conducted on D16.5 in mice of three groups (chow group, HFD group, treated group) to evaluate the role of chemerin in the glucose tolerance of HFD mice. IPGTT results showed that compared with the chow group, the blood glucose levels in the HFD group were explicitly heightened at 60 min and 90 min after glucose injection, while the treatment of chemerin could lessen the blood glucose levels of HFD mice at 120 min after glucose injection (Fig. 6A). Besides, chemerin treatment considerably reduced the weight of fetuses of HFD mice (Fig. 6B).

ELISA analysis showed a higher serum level of chemerin in the HFD group compared with the chow group, and the expression of chemerin was further elevated in the treated group (Fig. 6E). However, there were no significant differences in the fasting insulin level of pregnant mice among three groups on D18.5 (Fig. 6F). To detect the changes of lipid metabolism and liver function of pregnant mice after HFD and chemerin treatment, biochemical analyses were carried out. Results showed that the fasting serum levels of ALT, LDL, TC and TG were all obviously boosted in HFD group compared with the chow group (Fig. 6G, J, K, L), and the treatment of chemerin could mildly downregulate their content, although the differences were not statistically significant.

### Chemerin upregulated the expressions of AKT and DsbA-L, elevated the activity of SOD in HFD mice placenta

**Fig. 7:**
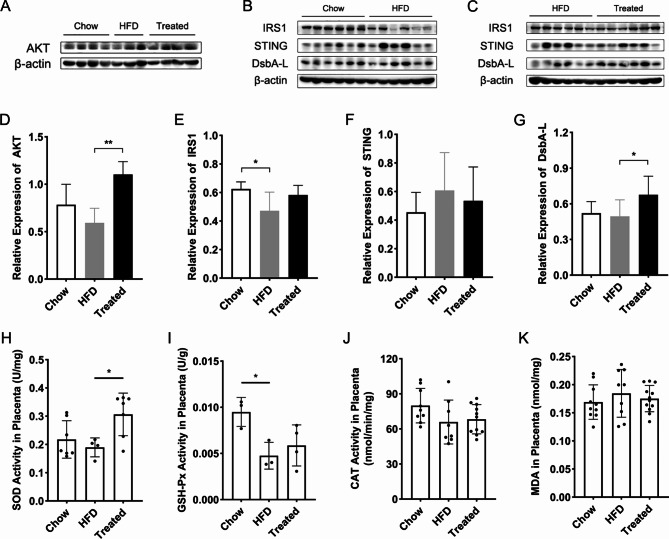
The roles and mechanisms of chemerin in the placental oxidative stress and insulin resistance in HFD pregnant mice. (**A**) Western blotting images of mice placentas of three groups. (**B**) Western blotting images of mice placentas between the chow groups and the HFD groups. (**C**) Western blotting images of mice placentas between the HFD groups and the treated groups. The HFD placenta samples presented in (**B**) and (**C**) were from the same several mice. (**D**) Protein quantification of AKT relative to β-actin. (**E**) Protein quantification of IRS1 relative to β-actin. (**F**) Protein quantification of STING relative to β-actin. (**G**) Protein quantification of DsbA-L relative to β-actin. (**H**) The activity of SOD in mice placenta. (**I**) The activity of GSH-Px in mice placenta. (**J**) The activity of CAT in mice placenta. (**K**) The content of MDA in mice placenta. HFD: high fat diet; AKT/PKB: protein kinase B; IRS1: insulin receptor substrate 1; STING: stimulator of interferon genes; DsbA-L: disulfide-bond A oxidoreductase-like protein; SOD: superoxide dismutase; GSH-Px: glutathione peroxidase; CAT: catalase; MDA: malondialdehyde. Data were shown as mean ± SD (*n* ≥ 3). ^*^P < 0.05; ^**^P < 0.01

To further evaluate the effects and mechanisms of chemerin on placental oxidative stress and insulin resistance in HFD pregnant mice, the protein expressions of insulin receptor substrate 1 (IRS1), AKT and DsbA-L as well as STING were detected by the experiment of western blotting, respectively. Results showed that compared to the HFD group, the expressions of AKT and DsbA-L were both considerably improved by chemerin treatment (Fig. 7A, C, D, G). In addition, the level of IRS1 of the HFD group was obviously declined compared with the chow group, whereas the treatment of chemerin gently heightened the level of IRS1 compared with the HFD group (Fig. 7B, C, E). However, there were no statistical differences in the expression of STING in three groups (Fig. 7B, C, F).

To examine the influences of chemerin on the placental oxidative stress of HFD pregnant mice, the activities of antioxidant enzymes, including SOD, GSH-Px, CAT, and the level of MDA were separately assayed. Results demonstrated that compared to the chow group, the activity of GSH-Px was markedly decreased in the HFD pregnant mice (Fig. 7I), and the activities of SOD and CAT of the HFD groups were also downregulated although the differences were not statistically significant (Fig. 7H, J). Notably, the treatment of chemerin played a vital role in elevating the activity of SOD of HFD pregnant mice placenta (Fig. 7H). Unfortunately, there was no significant difference in the content of MDA among the three groups (Fig. 7K).

### Chemerin could improve the overgrowth of HFD mice offspring between the ages of 1 and 4 weeks

**Fig. 8:**
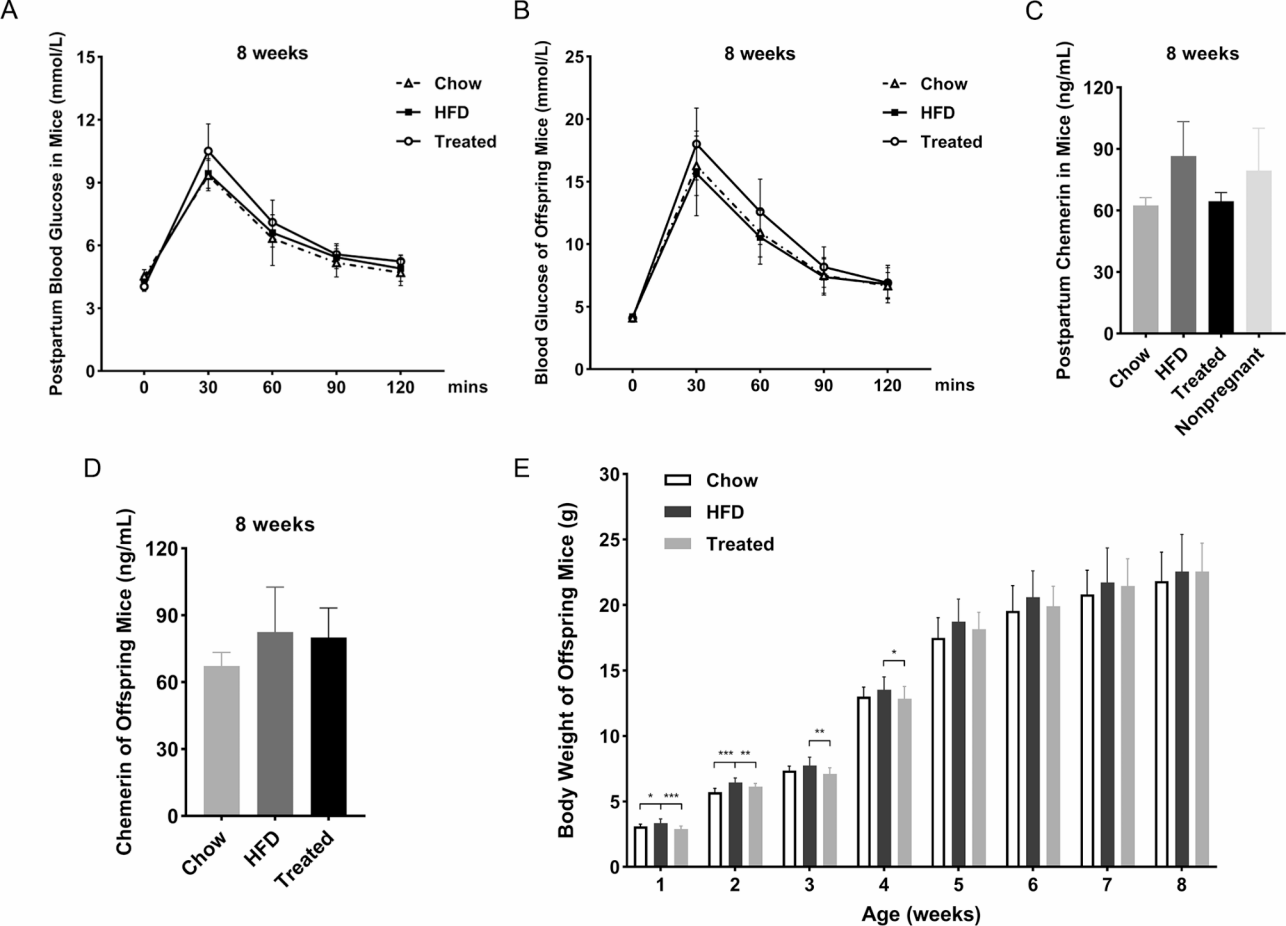
The long-term impacts of chemerin on blood glucose levels and body weight of maternal and fetal mice. (**A**) The postpartum blood glucose levels of the maternal mice at 8 weeks by IPGTT. (**B**) The blood glucose levels of the mice offspring at 8 weeks old by IPGTT. (**C**) The serum levels of chemerin in the maternal mice at 8 weeks after delivery. (**D**) The serum levels of chemerin in the mice offspring at 8 weeks old. (**E**) The changes of body weight of the mice offspring from 1 to 8 weeks of age. HFD: high fat diet. Data were shown as mean ± SD (*n* ≥ 3). ^*^P < 0.05; ^**^P < 0.01; ^***^P < 0.001

To evaluate the long-term influences of HFD and treatment of active chemerin on the blood glucose levels of mice and the body weight of offspring, several maternal mice and their offspring were fed continuously for 8 weeks after delivery by chow diet. The maternal and fetal glucose tolerance were detected by IPGTT, and the serum levels of chemerin were observed by ELISA. Results of IPGTT showed that there were no statistically significant differences in the postpartum blood glucose levels of maternal mice at 8 weeks in three groups (Fig. 8A), suggesting that the blood glucose level of GDM mice induced by HFD returned to normal after delivery and the intervention of chemerin did not have a long-term effect on blood glucose levels of mice. The ELISA results showed that there were also no statistically significant differences in the serum levels of chemerin among the three groups of maternal mice at 8 weeks after delivery. When combined with the chemerin levels of pregnant mice on D18.5, we found that the content of chemerin was significantly increased under the gestational state, and then decreased to the pre-pregnancy level at 8 weeks after delivery (Fig. 8C).

During the period of feeding mice offspring, the change of body weight was recorded once a week (Fig. 8E). Results showed that the body weight of HFD mice offspring was markedly elevated at the age of 1 week and 2 weeks, compared with those of chow mice offspring. Besides, in comparison with the HFD group, the body weight of mice offspring of the treated group was notably declined at the age of 1 ~ 4 weeks, which indicated that treatment of chemerin could reverse the phenotype of obesity induced by HFD. However, there was no significant difference in the body weight of mice offspring in three groups at the age of 5 ~ 8 weeks. Neither the glucose tolerance nor serum levels of chemerin were found statistically significant differences among mice offspring at 8 weeks old (Fig. 8B, D).

## Discussion

GDM was defined as hyperglycemia firstly recognized during pregnancy (American Diabetes Association 2024). Oxidative stress, attributed to the imbalance between ROS generation and elimination, was implicated in the pathology of GDM. The high glucose microenvironment within the placenta of GDM could lead to oxidative stress and mitochondrial dysfunction, both of which are significant contributors to the progression of insulin resistance and metabolic disorders (Fisher et al. 2020). Studies have found that ROS are normally mitigated by antioxidant enzymatic systems, including SOD, GSH-Px, CAT (Hu and Zhang 2021). The accumulation of ROS can cause oxidative damage to lipids, and MDA as well as LPO often occur as the vital products of peroxidation. In the present study, the observed mild elevation in the activities of SOD, GSH-Px and CAT enzymes in GDM placenta were possibly an adaptive response to elevated levels of ROS and oxidative stress (Fisher et al. 2021). The level of LPO that represented total lipid peroxidation products was significantly higher, and glutathione was seriously oxidized in GDM placenta compared with those of the normoglycaemic pregnant women. Our findings indicated an association between GDM and oxidative stress, in concordance with the previous study (Zaugg et al. 2024). Pregnancy itself was accompanied with persistent oxidative stress, while GDM could aggravate this condition and result in mitochondrial disruption (Hebert and Myatt 2021).

During pregnancy, placenta acts as an endocrine organ and secretes numbers of cytokines and adipokines, including chemerin, to get involved in the maternal metabolism and fetal growth and development (Ruszała et al. 2021). Chemerin was originally discovered as a chemokine, implying an immunomodulating role. Over the past decade, it was characterised as a novel functional adipokine that regulated adipogenesis, glucose homeostasis, body metabolism and energy balance (Helfer and Wu 2018), however, contradictory results were reported on the association of chemerin with insulin signaling and glucose metabolism (Gutaj et al. 2020). The discrepancy in various researches might be attributed to the different concentrations, durations of chemerin application and culture conditions. Previous studies have described that there were insulin resistance and oxidative stress in the high glucose cell model (Zhou et al. 2023). Based on that, our current findings further established that chemerin had a distinct role for stimulating glucose uptake, enhancing antioxidant capacity and regulating the ROS levels. Previous work has preliminarily revealed the regulation of chemerin on cGAS-STING signaling pathway in the cultured human trophoblast cell lines (Zhou et al. 2023), yet additional animal experiments are needed to identify the role of the chemerin in placental oxidative stress in the context of GDM.

In the GDM mouse model, we found obviously elevated expression of chemerin and declined level of DsbA-L in placenta, these findings were agreed with our previous clinical study (Zhou et al. 2023). The decreased expression of DsbA-L indirectly revealed the damaged mitochondrial function. It is interesting to note that with the results of up-regulation of TBK1 and IRF3 downstream of cGAS-STING signaling pathway, we provided evidence that this pathway was activated in GDM placenta. Previous work has highlighted that cGAS-STING pathway is located at the crossroads of inflammation and metabolism (Gong et al. 2024). The activation of cGAS-STING pathway might further inhibit insulin signaling and promote inflammatory and oxidative stress (Oduro et al. 2022). Moreover, ultrastructural microscope presented the morphological changes in mitochondria from the HFD mice placenta, which was a hallmark of mitochondrial dysfunction. Previous study had found a strong association between mitochondrial structure and function. Mitochondria will overproduce ROS if their morphology undergo changes, causing further mitochondrial damage and forming a vicious cycle (Hu and Zhang 2021).

To investigate the effect of chemerin on blood glucose in vivo, HFD mice were treated by active recombinant chemerin. Results showed that exogenous treatment further stimulated the secretion of endogenous chemerin, and it decreased the blood glucose level at 120 min of IPGTT, which might be related to the potentiation of insulin-dependent glucose uptake (Zorena et al. 2020). It has been reported that chemerin played a significant role in promoting glucose clearance and adapting glucose metabolism to alteration in dietary fat (Fang et al. 2021). To the best of our knowledge, hyperglycemia is often accompanied with abnormal lipid metabolism, as indicated by the considerably elevated serum levels of TC, TG and LDL in HFD mice. Results also showed that the liver of HFD mice had many fat vacuoles and lipid droplets, and the level of ALT was heightened due to liver steatosis and dysfunction. The active chemerin treatment was favorable for lessening the blood lipid level and ameliorating liver functions, although its effects were not very substantial.

Notably, the expression of chemerin was found to be significantly increased in serum of HFD mice relative to normal pregnant mice, possibly as a compensatory response to glucose metabolic disorders. However, we did not distinguish the various isoforms of chemerin due to the restriction of currently commercially available chemerin ELISA kits (Zhao et al. 2024). Further exploration by using the mass spectrometry analysis or antibodies against specific chemerin isoforms will likely lead to discovery of the differential roles of active and inactive chemerin. Reassuringly, the present study provided the evidence, specifically from in vivo assays, that chemerin might modulate the insulin signaling by up-regulating the expression of AKT in placenta of GDM mice. Importantly, chemerin treatment enhanced the expression level of DsbA-L, thereby probably alleviating mitochondrial dysfunction and insulin resistance in HFD mice (Yang et al. 2024). In contrast with the chow group, the activity of GSH-Px in the placenta of HFD mice markedly decreased, reflecting the insufficient antioxidant capacity of the placenta. Nevertheless, chemerin might attenuate the oxidative stress level of HFD mice placenta by increasing the activity of SOD (Hebert and Myatt 2021). Compelling data have demonstrated that vitamin A could increase the activities of mitochondrial complexes I and II through its metabolite, retinoic acid (RA) (Hebert and Myatt 2021; Blaner et al. 2021). Chemerin is coded by *RARRES2* gene, which has been identified as RA receptor responder and could be regulated by RA (Tan et al. 2023). Therefore, RA is a potent inducer of chemerin, this association may be an important factor underlying the reason for chemerin improving oxidative stress. However, further mechanistic studies are needed to dissect the molecular basis in detail.

In the present study, we found that the body weight of HFD mice offspring at 1 week and 2 weeks of age was remarkably increased compared with that of the chow group, which might be related to the overproduction of endogenous fetal insulin and insulin-like growth factor 1 stimulated by glucose (Yin et al. 2022). By contrast, reduced body weight of offspring from birth to 4 weeks old was observed in HFD mice treated with chemerin, suggesting that chemerin could reverse fetal overgrowth induced by HFD. This may be ascribed to the roles of chemerin in promoting glucose metabolism and reducing blood glucose level in HFD mice. The differences in body weight gradually disappeared as the mice offspring grew older. At 8 weeks after delivery, the content of chemerin in serum of maternal mice recovered to the pre-pregnancy level, and there was no statistical difference in blood glucose between different groups of mice and its offspring, which confirmed that our GDM model presented transient changes in glucose intolerance during pregnancy, and also indicated that chemerin administration had no long-term effect on the blood glucose of mother and child. Future studies are required to verify whether chemerin has other beneficial effect on HFD mice offspring.

However, our research has several limitations which need to be highlighted. Additional details about the precise molecular mechanisms through which chemerin regulates oxidative stress need to be further elucidated. In addition, knocking out or silencing the gene of chemerin would have made the present results more convincing. It will be interesting to explore the improvement of mitochondrial function by detecting the dynamic changes of mitochondria in the future.

## Conclusions

In summary, combining the current data, we demonstrated that chemerin could play a vital role in ameliorating oxidative stress and hyperglycemia, resulting in improved fetal overgrowth. Our findings not only confirmed and extended the potential biology of chemerin, but also provided novel insights into intervention targets in GDM.

## Data Availability

No datasets were generated or analysed during the current study.

## Abbreviations

AKT/PKB: Protein kinase B
ALT: Alanine aminotransferase
AOD: Average optical density
AST: Aspartate transaminase
BMI: Body mass index
CAT: Catalase
cGAS: Cyclic GMP-AMP synthase
DsbA-L: Disulfide-bond A oxidoreductase-like protein
ELISA: Enzyme-linked immunosorbent assay
FINS: Fasting insulin
GDM: Gestational diabetes mellitus
GSH-Px: Glutathione peroxidase
GSSG: Oxidized glutathione
HDL: High-density lipoprotein
HE: Hematoxylin eosin
HFD: High-fat diet
HG: High glucose
IPGTT: Intraperitoneal glucose tolerance test
IRF3: Interferon regulatory factor 3
IRS1: Insulin receptor substrate 1
JZ: Junctional zone
LA: Labyrinth
LDL: Low-density lipoprotein
LPO: Lipid peroxidation
MDA: Malondialdehyde
OGTT: Oral glucose tolerance test
PI3K: Phosphatidylinositol 3-kinase
RA: Retinoic acid
RARRES2: Retinoic acid receptor responder 2
ROS: Reactive oxygen species
SOD: Superoxide dismutase
STING: Stimulator of interferon genes
T-AOC: Total antioxidant capacity
TBK1: TANK binding kinase 1
TC: Total cholesterol
TEM: Transmission electron microscope
TG: Triglyceride
